# Guaranteeing QoS for NOMA-Enabled URLLC Based on *κ*–*μ* Shadowed Fading Model

**DOI:** 10.3390/s22145279

**Published:** 2022-07-14

**Authors:** Jie Zeng, Yuxin Song, Teng Wu, Tiejun Lv, Shidong Zhou

**Affiliations:** 1 School of Communication and Information Engineering, Chongqing University of Posts and Telecommunications, Chongqing 400065, China; s200131222@stu.cqupt.edu.cn (Y.S.); s200131102@stu.cqupt.edu.cn (T.W.); 2 Beijing National Research Center for Information Science and Technology, Tsinghua University, Beijing 100084, China; zhousd@tsinghua.edu.cn; 3 School of Information and Communication Engineering, Beijing University of Posts and Telecommunications, Beijing 100876, China; lvtiejun@bupt.edu.cn; 4 Department of Electronic Engineering, Tsinghua University, Beijing 100084, China

**Keywords:** cell-free massive multiple-input multiple-output (CF mMIMO), κ–μ shadowed fading model, statistical delay quality-of-service (QoS), ultrareliable and low latency communications (URLLC)

## Abstract

Sixth-generation (6G) wireless communication scenarios are complex and diverse. Small-scale fading is a key part of wireless channels and its impact on performance in scenarios with time sensitivity and 6G ultrareliable and low latency communications (URLLC) quality-of-service requirements cannot be ignored. Therefore, it is necessary to accurately characterize small-scale fading when designing wireless communication systems. In this paper, we derive approximate closed form expressions for the probability density function, cumulative distribution function and moment-generating function of the postprocessing signal-to-noise ratio following the zero-forcing detector in a cell-free massive multiple-input multiple-output (CF mMIMO) system. CF mMIMO system is a nonorthogonal multiple access (NOMA) system that enables users to share all channel uses and can ensure the fairness of the communication quality experienced by different users. Our key contributions include the extension of the κ–μ shadowed fading model to a CF mMIMO system and the proposal of theoretical tools (the derived closed-form expression) to improve its mathematical tractability. By exploiting the statistical characterizations of the arrival and service processes, another important contribution is the exploitation of the upper bound of the queuing delay violation probability (UB-QDVP) over the Mellin transforms of the arrival and service processes in the proposed CF mMIMO system under the κ–μ shadowed fading model. Corroborated by extensive simulations, our analyses validate that the CF mMIMO system outperforms the orthogonal multiple access and power-domain NOMA systems and reveal the relationships among different small-scale fading types, energy efficiency, delay and the UB-QDVP, as well as the accuracy and effectiveness of the proposed theoretical tools based on the κ–μ shadowed fading model.

## 1. Introduction

### 1.1. Background and Motivation

Due to the highly time-varying nature of wireless channels, it is difficult to design communication systems that support ultrareliable and low latency communications (URLLC) with reductions in energy consumption. For example, the explosively increasing volumes of delay-sensitive and bandwidth-intensive applications under stringent quality-of-service (QoS) requirements have required strict delay (<0.1 ms), ultrareliability (>99.99999%), and extra-high energy efficiency (EE) demands [1]. Notably, achieving higher data rates and lowering power consumption seem to be contradictory objectives. In particular, when the delay boundary approaches the channel coherence time, the transmit power required to obtain the QoS requirements becomes unbounded [2]. Although both academia and industry have paid attention to the EE of cellular networks in the past few years [3], the related works are still unable to address the increasing complexity of devices, antennas and many frequency bands in the future [4].

Orthogonal multiple access (OMA) systems (e.g., multiple-input multiple-output orthogonal frequency division multiple access (MIMO-OFDMA)) apply the orthogonal resource allocation method, in which different uplink users occupy different channel uses (CUs). This type of system distinguishes different users through different orthogonal time–frequency resource blocks. However, all users share time–frequency resources in the non-OMA (NOMA) system. As a promising technology, NOMA makes full use of the nonorthogonal nature over limited orthogonal resources so as to provide a higher transmission accuracy with lower computational complexity [5], and to meet the massive connectivity requirements in an Internet of things scenario [6,7] and even satellite-based communications [8]. For instance, power-domain NOMA (PD-NOMA), considered as the representative of single carrier NOMA technology [9], distinguishes different groups of users through the spatial domain, and different users in the same group share spatial domain resources. Under PD-NOMA systems, power control is required to meet the QoS requirements of URLLC for energy efficiency, latency and reliability [10]. However, fairness among the users in terms of their throughput is an important goal of NOMA power allocation [11]. For the upcoming sixth generation (6G) mobile networks, a beneficial embodiment of a distributed massive MIMO system is the cell-free massive MIMO (CF mMIMO) system proposed in [12]. As a type of NOMA system, the CF mMIMO system is a better solution between PD-NOMA and MIMO-OFDMA. All users share all CUs, and different users are distinguished by the spatial domain without considering the fairness experienced by uplink users. The CF mMIMO system contains a large number of randomly distributed access points (APs) to coherently serve massive mobile devices; its implementation is more feasible for practical issues than network MIMO with low overhead that exploits channel hardening phenomena and favorable propagation properties [4,13].Relative to traditional cellular mMIMO networks, the notable characteristics of CF mMIMO system are its superior macro diversity and interference suppression capabilities. Macro diversity offers orders of magnitude of coverage probability [12,14], reliability and delay optimization through suitable signal processing. Intercell interference is effectively mitigated by the user-centric approach and coordination [15,16], thereby improving the accuracy of multiuser detection. To the best of our knowledge, a CF mMIMO system that efficiently integrates the unified fading model to provide substantial performance improvements with respect to different types of small-scale fading has still not been well studied.

On the other hand, most current papers concerning the performance analysis in CF mMIMO systems [4,17,18,19] perform analyses in the context of small-scale fading models such as the Rayleigh and Nakagami-*m* models, which ignore the random nature of wireless fading channels. Such classic fading models usually do not cover all types of fading scenarios encountered in practical cases. More importantly, the analyses performed for practical channel estimations depend on the channel’s statistical properties, which are affected by the selected channel models [20]. Consequently, many results are not reasonably universal enough due to simplified assumptions, such as in many studies of CF mMIMO systems performance based on the Rayleigh model [12,14,21] or Rician model [22]. The design of reliable wireless communication systems is highly dependent on the accurate characterization of the wireless fading channel. However, for the complex communication scenarios in future 6G URLLC scenarios [23,24], the classic fading model is no longer applicable [25,26]. With the research on fading models in recent years, a new fading model that can losslessly portray a variety of fading types has the potential to become a unified fading model [27,28,29,30], which can be used to accurately characterize fading channels in complex future 6G URLLC scenarios. This is a critical issue regarding the theoretical analysis and performance optimization of URLLC, given that the reliability of modeling is of capital importance. Specifically, the κ–μ shadowed fading model, which unifies the κ–μ fading distribution and the η–μ fading distribution, was introduced in [27]. First, by adjusting the parameters κ, μ and *m*, almost all classical fading models can be unified by the κ–μ shadowed fading model [28] with similar mathematical flexibility levels. Second, this shadowed model provides much better tractability and flexibility [31]. These two features allow the κ–μ shadowed fading model to accommodate different propagation conditions, such as in practical communication environments that experience the effects of nonhomogeneous fading conditions. However, although the κ–μ shadowed fading model can characterize a variety of fading channels, adapting the κ–μ shadowed fading model to the CF mMIMO system and specifically analyzing the system performance while supporting URLLC becomes a challenging research issue.

With respect to mathematical tools, towards the exponentially growing demands of time-sensitive services, the traditional Shannon information theory based on the infinite blocklength assumption and a random coding scheme provides a poor benchmark in this scenario [32]. Finite-blocklength (FBL) information theory was proposed in [33] as a short-packet data communication technique for guaranteeing the stringent QoS requirements of URLLC [34]. Well-known results regarding the maximum achievable data rate of FBL packets were derived in [33] as a function of the blocklength and error probability. Moreover, as suggested by Bennis et al. [35] and Arnau et al. [36], the design of URLLC should capture the tail behavior of reliability (i.e., queuing delay violation probability) and delay instead of average metrics. Because the delay and overall reliability consist of multiple components, the corresponding performance indicators should be constrained by a delay boundary and a delay violation probability boundary for URLLC [36]. In this sense, the stochastic network calculus (SNC) approach proposed in [37] provides a nonasymptotic boundary on the delay violation probability as an effective analysis tool for connecting the delay and overall reliability.

Therefore, the CF mMIMO system under the κ–μ shadowed fading model is sufficient for analyzing performance in practical communication environments to meet the strict QoS requirement of URLLC. However, a major problem with this kind of performance analysis is appropriately characterizing stochastic queuing behaviors when they are integrated with the CF mMIMO system under the κ–μ shadowed fading model with SNC and FBL information theory. Furthermore, it is still an urgent issue to exploit the effect of small-scale fading characteristics on the performance of the CF mMIMO system under the κ–μ shadowed fading model to achieve NOMA-enabled URLLC with high resource efficiency.

### 1.2. Related Works

Our paper builds on results derived from several research areas. On the physical layer, we consider the uplink CF mMIMO system with imperfect channel state information (CSI) under the κ–μ shadowed fading model as well as FBL information theory. On top of a service description in the physical-layer fading channel model, we then investigate service processes. We determine that the effect of different types of small-scale fading on the performance in the design, modeling and analysis of several wireless communication systems cannot be neglected.

The CF mMIMO system has been gradually considered in the URLLC scenario. Nasir et al. [38] first investigated a CF mMIMO system for downlink URLLC in the FBL regime and designed a special class of conjugate beamforming to achieve a lower computational complexity and improved URLLC rate performance. Elwekeil et al. [19] introduced power control schemes in a CF mMIMO system that supported URLLC applications for both traditional ground users and unmanned aerial vehicles, and they evaluated the superior rate performance of this CF mMIMO system. Due to the spatial domain, the CF mMIMO method can be a key to boosting the reliability of URLLC applications. The study in [39] compared different transmission modes in a factory automation scenario and demonstrated that the user-centric transmission mode and power control brought substantial performance improvements in terms of reliability and latency. Zhang et al. [40] integrated the CF mMIMO approach with simultaneous wireless information and power transfer for statistical delay and error-rate bounded QoS to support URLLC and boost the data rate and energy efficiency. Hence, a CF mMIMO network can be viewed as a promising system to support URLLC with a high resource efficiency and low latency.

The κ–μ shadowed fading model [27,28] was proposed as a more general yet equally tractable model to capture a wide range of propagation conditions with clear physical interpretation and good analytical properties. Furthermore, Lopez-Martinez et al. [31] derived the probability density function (PDF) and cumulative distribution function (CDF) of the κ–μ shadowed fading model with integer fading parameters. Ramirez-Espinosa et al. [25] provided a statistical characterization of the κ–μ shadowed fading model and asserted that these results could be used to derive some performance metrics of wireless communication systems over fading channels. With respect to the characterization of the κ–μ shadowed fading model, its applications have been studied in several works. ElHalawany et al. [41] carried out a performance (i.e., ergodic capacity, outage probability and average bit error rate) analysis of downlink NOMA systems subject to the κ–μ shadowed fading model. Chun et al. [42] proposed an analytic framework to evaluate the average of an arbitrary signal-to-noise-plus-interference ratio (SINR) function over the κ–μ shadowed fading model and evaluate the spectral efficiency, moments of the SINR and outage probability of heterogeneous cellular systems. The authors in [43] characterized the impacts of realistic propagation conditions on the achievable secrecy performance of MIMO systems over the κ–μ shadowed fading model. Fully absorbing the attractive nature of the κ–μ shadowed fading model is seen as one of the potential keys to characterizing the propagation media in emerging practical scenarios.

Several works have also examined SNC-based network performance analysis and theoretical boundary calculation for URLLC. Schiessl et al. used SNC to investigate the delay performance of a multiuser MIMO system with zero-forcing beamforming [44] and NOMA with joint decoding [45] under imperfect CSI and FBL. Xiao et al. [46] derived a closed-form of the upper bound of the queuing delay violation probability (UB-QDVP) in a downlink NOMA system and designed an optimal power allocation scheme for guaranteeing the delay violation probability. Furthermore, by utilizing moment generating function (MGF)-based SNC, probabilistic delay bounds for traffic dispersion, network densification and a hybrid scheme were obtained in millimeter-wave communications over Nakagami-*m* fading [47]. A study close to our work is the research by Zhang et al. [22], where the stochastic QoS performance in terms of both the delay and error rate metrics was obtained for a downlink CF mMIMO based system model across Rician fading in the FBL regime. However, only traditional fading channels have been considered for discussion in the related literature, while no investigation is available with respect to the performance analysis for CF mMIMO systems with the juxtaposition of shadowing and small-scale fading.

### 1.3. Main Contributions

For 6G URLLC requirements, we combine the potential key technology of future 6G, CF mMIMO [48], to build the uplink wireless communication system, and extend the κ–μ shadowed fading model to the CF mMIMO system, for lossless characterization of its fading channel in the complex communication scenario of 6G [23,24]. The key contributions of the paper can be summarized as follows.

- We derive approximate closed-form expressions for statistical characteristics (the PDF, CDF and MGF) of the sum of independent and nonidentically distributed (i.n.i.d.) κ–μ shadowed random variables (RVs). The analysis is nontrivial, as we optimize the theoretical analysis performance of the κ–μ shadowed fading model and improve its mathematical tractability.
- Based on the κ–μ shadowed fading model, we derive approximate closed-form expressions for the PDF, CDF and MGF of the postprocessing signal-to-noise ratio (PPSNR) after the zero-forcing detector in the proposed CF mMIMO system, and extend the κ–μ shadowed fading model to 6G NOMA wireless communication systems.
- By utilizing FBL information theory, SNC and the Mellin transform on the service process, we exploit the UB-QDVP in the proposed CF mMIMO system under the κ–μ shadowed fading model. Furthermore, based on extensive simulations, we analyze the system performance with the delay and UB-QDVP indicators and validate the necessity of analyses performed under the κ–μ shadowed fading model; the CF mMIMO system outperforms the OMA system and PD-NOMA system. (For the OMA and PD-NOMA systems, there are *L* antennas equipped in their base station and *K* users. For illustration convenience, in the PD-NOMA system, we divide *K* users into N pairs based on the channel gains of the users (K=2N). Specifically, we divide *K* users into a group of N “strong users” and a group of N “weak users” according to their channel gains and sort the users in the descending order of their channel gains within each of the groups. The “strong user” and “weak user” with the same label are grouped in the same pair.)

### 1.4. Organization

The remainder of this paper is organized as follows. In Section 2, the system model for a CF mMIMO system under the κ–μ shadowed fading model is described. Section 3 introduces SNC and presents the Mellin transform over both arrival and service processes as well as the UB-QDVP. Simulation results and an analysis are given in Section 4, followed by the conclusions in Section 5.

*Notations*: $C^{K\times N}$ is a complex matrix with *K* rows and *N* columns. The modulus and expectation operators are denoted by $\left|•\right|$ and E(•), respectively. ${\left(•\right)}^{T}$ and ${\left(•\right)}^{H}$ denote the transpose and conjugate transpose, respectively, of their arguments. ${\left(•\right)}_{lk}$ denotes the element in the *l*th row and *k*th column of a matrix. CN(0,1) denotes a complex Gaussian distribution with a mean of zero and unit variance.

## 2. System Model

**Figure 1:**
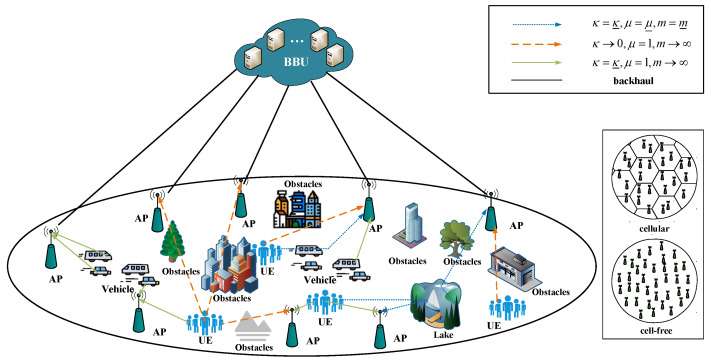
CF mMIMO system under the κ–μ shadowed fading model.

We consider an uplink CF mMIMO system in which *L* single-antenna APs and *K* single-antenna users are randomly uniformly distributed in a circular area with L≫K, as shown in Figure 1. Some conventional fading can be obtained by adjusting the parameters of the κ–μ shadowed fading model: Rayleigh fading (κ→0,μ=1,m→∞), Rician fading ($\kappa \;=\;\underline{\kappa }$, *μ* = 1, m→∞), Rician shadowed fading ($\kappa \;=\;\underline{\kappa }$, *μ* = 1, $m=\underline{m}$), and other fading ( $\kappa \;=\;\underline{\kappa }$, $\mu =\underline{\mu }$, $m=\underline{m}$, where $\underline{\kappa }$, $\underline{\mu }$ and $\underline{m}$ are nonnegative real numbers. When $\underline{m}\to \infty$, it denotes that no shadow effect is observed). All APs simultaneously serve all users at the same time–frequency resource and are connected to a baseband unit (BBU) for centralized signal processing via ideal backhaul links [19,49] (There is no delay in the ideal backhaul links between APs and the BBU [50]). The transmission from the APs to the users (downlink transmission) and the transmission from the users to the APs (uplink transmission) proceed by a time-division-duplexing operation. Each coherence interval is divided into three phases: uplink training, downlink payload data transmission, and uplink payload data transmission. In the uplink training phase, the users send pilot sequences to the APs and the BBU estimates the channel for all users. In this paper, the QoS delay for URLLC is characterized by the queuing delay (Considering ideal backhaul links and tail behaviors based on [35,36], we consider the queuing delay as the delay of the URLLC QoS requirement). Each user transmits a short packet with *D* information bits through *N* CUs, which are spread over *B* MHz of bandwidth and $t_{f}$ milliseconds of time ($N=Bt_{f}$).

The channel coefficient $g_{lk}\left(t\right)$ between the *l*th AP and the *k*th user can be characterized as

(1) $$g_{lk}\left(t\right)=\sqrt{\beta_{lk}\left(t\right)}h_{lk}\left(t\right),$$

where $\beta_{lk}\left(t\right)$ is a large-scale fading coefficient that changes very slowly with time and is assumed to be constant over many coherence conditions. $h_{lk}\left(t\right)$ is the small-scale fading coefficient of the corresponding links, which is assumed to be static during a finite-sized time–frequency coherence interval, and it changes independently from one coherence interval to the next [51]. The channel response is assumed to follow the κ–μ shadowed fading model. The gain of small-scale fading between the *k*th ($k\in \left[1,K\right]$) user and the *l*th AP ($l\in \left[1,L\right]$) can be modeled as

(2) $${\left|h_{lk}\right|}^{2}=\sum_{i=1}^{\mu_{lk}}{\left(X_{lk,i}+\xi_{lk}{\hat{p}}_{lk,i}\right)}^{2}+{\left(Y_{lk,i}+\xi_{lk}{\hat{q}}_{lk,i}\right)}^{2},$$

where $\mu_{lk}$ is a natural number. Each multipath cluster is modeled by one term of the sum; thus, $\mu_{lk}$ is the number of multipath clusters. Each cluster has scattered components with the same power and dominant components with a certain arbitrary power. $X_{lk,i}$ and $Y_{lk,i}$ are independent and identically distributed (i.i.d.) Gaussian RVs with means of zero and variances of $\sigma^{2}$, and $X_{lk,i}+jY_{lk,i}$ denotes the scattered components of the *i*th cluster. ${\hat{p}}_{lk,i}$ and ${\hat{q}}_{lk,i}$ are real numbers and the mean values of the in-phase and quadrature parts of the multipath components of the *i*th cluster, respectively. $\xi_{lk}$ is a Nakagami-*m* RV with shaping parameter and $E\left[\xi_{lk}^{2}\right]=1$, where ξlk2∼Γmlk,11mmlk; $m_{lk}$ represents the fluctuation degree of dominant components caused by the shadow [28]. For each cluster, the total power of the scattered components is $2\sigma^{2}$. Furthermore, the ratio of dominant components to scattered components in small-scale fading is $\kappa_{lk}=\frac{\sum_{i=1}^{\mu }{\hat{p}}_{lk,i}^{2}+{\hat{q}}_{lk,i}^{2}}{2\mu_{lk}\sigma^{2}}$. In addition, ${\left|h_{lk}\right|}^{2}$ statistically follows the κ–μ shadowed distribution, whose PDF can be expressed approximately by the Gamma distribution, namely, ${\left|h_{lk}\right|}^{2}∼\Gamma \left(K_{lk},\theta_{lk}\right)$, where Klk=mlkμlk1+κlk2mlk+μlkκlk2+2mlkκlk=μlk1+κlk21+μlkκlk2μlkκlk2mmlk+2κlk; $\theta_{lk}=\frac{\Omega_{lk}}{K_{lk}}$; $\Omega_{lk}$ is the mean value of ${\left|h_{lk}\right|}^{2}$.

The received signal at the BBU under imperfect CSI can be denoted as in the following equation.

(3) $$\left[Y^{\left(p\right)}\left(t\right),Y^{\left(d\right)}\left(t\right)\right]=\sqrt{p_{u}}G\left(t\right)\left[X^{\left(p\right)}\left(t\right),X^{\left(d\right)}\left(t\right)\right]+\left[Z^{\left(p\right)}\left(t\right),Z^{\left(d\right)}\left(t\right)\right],$$

where $p_{u}$ is the average transmit power of each user. $Y^{\left(p\right)}\left(t\right)\in C^{L\times n}$ and $Y^{\left(d\right)}\left(t\right)\in C^{L\times \hat{n}}$ are received pilot signal and received data signal transmitted by the users at time slot *t*, respectively. *n* and $\hat{n}$ are the length of the pilots (LoP) and the length of the data signal, respectively, which satisfy $n+\hat{n}=N=Bt_{f}$. $X^{\left(p\right)}\left(t\right)={\left[x_{1}^{\left(p\right)}\left(t\right),\ldots ,x_{K}^{\left(p\right)}\left(t\right)\right]}^{T}\in C^{K\times n}$ is the transmitted pilot signal at slot *t*, which satisfies $X^{\left(p\right)}\left(t\right){\left(X^{\left(p\right)}\left(t\right)\right)}^{H}=nI_{K}$. $X^{\left(d\right)}\left(t\right)={\left[x_{1}^{\left(d\right)}\left(t\right),\ldots ,x_{K}^{\left(d\right)}\left(t\right)\right]}^{T}\in C^{K\times \hat{n}}$ is the transmitted data signal at time slot *t*, obtained by assuming that the elements of $X^{\left(d\right)}\left(t\right)$ are not related to each other, namely $E\left({\left|x_{k}^{\left(d\right)}\left(t\right)\right|}^{2}\right)=n$, $E\left({\left(x_{k}^{\left(d\right)}\left(t\right)\right)}^{T}x_{j}^{\left(d\right)}\left(t\right)\right)=0$, $\forall k,j\in \left[1,K\right]\;\mathrm{and}\;k\neq j$. $G\left(t\right)=\left[g_{1}\left(t\right),\ldots g_{K}\left(t\right)\right]\in C^{L\times K}$ is the CSI of *K* users and *L* APs, where $g_{k}\left(t\right)={\left[g_{1k}\left(t\right),\ldots g_{Lk}\left(t\right)\right]}^{T},\forall k\in \left[1,K\right]$. As we mentioned above, the expression for the channel coefficient is $g_{lk}\left(t\right)=\sqrt{\beta_{lk}\left(t\right)}h_{lk}\left(t\right)$, $\forall k\in \left[1,K\right]$, and $\forall l\in \left[1,L\right]$, which can be simplified as $g_{lk}=\sqrt{\beta_{lk}}h_{lk}$, $g_{k}\left(t\right)\equiv g_{k}$ and $G\left(t\right)\equiv G$ because the large-scale fading coefficient $\beta_{lk}\left(t\right)$ and small-scale fading coefficient $h_{lk}\left(t\right)$ are generally assumed to be RVs that are independent of time. $Z^{\left(p\right)}\left(t\right)$ and $Z^{\left(d\right)}\left(t\right)$ represent additive white Gaussian noise (AWGN), whose elements are i.i.d.; this can be expressed as CN(0,1). In a practical communication system, serving users with a large number of APs requires accurate CSI to be available. The estimated channel matrix $\hat{G}$ obtained via the least squares estimation method which relies on the received signal with the known pilot sequence is expressed as [52]

(4) $$\hat{G}=\Delta G+\hat{E},$$

where $\hat{G}=\left[{\hat{g}}_{1},\ldots {\hat{g}}_{K}\right]\in C^{L\times K}$, $\Delta ={\left[\Lambda_{1},\ldots ,\Lambda_{K}\right]}^{T}\in C^{K\times L}$, $\Lambda_{k}={\left[\Lambda_{1k},\ldots ,\Lambda_{LK}\right]}^{T}$ and $\Lambda_{lk}=\sqrt{\frac{np_{u}\beta_{lk}\Omega_{lk}}{1+np_{u}\beta_{lk}\Omega_{lk}}}$. $\beta_{lk}$ and $\Omega_{lk}$ are the large-scale fading coefficient and mean square value of the small-scale fading coefficient, respectively. $\hat{E}=\left[{\hat{e}}_{1},\ldots ,{\hat{e}}_{K}\right]\in C^{L\times K}$ is the channel estimation error matrix, and its elements ${\hat{e}}_{k}={\left[{\hat{e}}_{1k},\ldots ,{\hat{e}}_{Lk}\right]}^{T}$ follow ${\hat{e}}_{lk}∼CN\left(0,\sigma_{{\hat{e}}_{lk}}^{2}\right)$, where $\sigma_{{\hat{e}}_{lk}}^{2}=\Lambda_{lk}^{2}\sigma_{e_{lk}}^{2}=\frac{\Lambda_{lk}^{2}}{np_{u}}=\frac{\beta_{lk}\Omega_{lk}}{1+np_{u}\beta_{lk}\Omega_{lk}}$. The elements of $\hat{G}$ can be expressed as ${\hat{g}}_{lk}=\Lambda_{lk}g_{lk}+{\hat{e}}_{lk}=\sqrt{\beta_{lk}}{\hat{h}}_{lk}$; thus, ${\hat{h}}_{lk}=\Lambda_{lk}h_{lk}+\frac{{\hat{e}}_{lk}}{\sqrt{\beta_{lk}}}$.

Adopting the standard linear zero-forcing detector $\hat{A}=\hat{G}{\left({\hat{G}}^{H}\hat{G}\right)}^{-1}\in C^{L\times K}$, the received signal of the *k*th user is

(5) $$R\left(t\right)=\sqrt{p_{u}}{\hat{A}}^{H}G\left(t\right)X^{\left(d\right)}\left(t\right)+{\hat{A}}^{H}Z^{\left(d\right)}\left(t\right).$$

After completing zero-forcing detection, the PPSNR of the *k*th user is [53]

(6) $$\begin{array}{cc} {\hat{\gamma }}_{k} & =\frac{p_{u}}{p_{u}\sum_{l=1}^{L}{\left|{\hat{a}}_{lk}\right|}^{2}\sum_{n=1}^{K}\sigma_{{\hat{e}}_{\ln}}^{2}+{∥{\hat{a}}_{k}∥}^{2}}\\ \; & \overset{\left(a\right)}{\approx }\frac{p_{u}}{\frac{K}{n}{∥{\hat{a}}_{k}∥}^{2}+{∥{\hat{a}}_{k}∥}^{2}}\\ \; & =\frac{p_{u}}{\left(\frac{K}{n}+1\right){∥{\hat{a}}_{k}∥}^{2}}\\ \; & \overset{\left(b\right)}{\approx }\frac{p_{u}}{\left(\frac{K}{n}+1\right)}\sum_{\hat{l}\in L_{k}}{\left|g_{\hat{l}k}\right|}^{2}\\ \; & ={\hat{p}}_{u}\sum_{\hat{l}\in L_{k}}\eta_{\hat{l}k}\\ \; & ={\hat{p}}_{u}\zeta_{k} \end{array},$$

where $L_{k}=L/B_{k}=\left\{\hat{l}\left|\forall \hat{l}=1,...,\hat{L}\right.\right\}$, $L=\left\{l\left|\forall l=1,...,L\right.\right\}$, $B_{k}$ is defined as $B_{k}\overset{\Delta }{\;=}\mathrm{Unique}\left(\left\{l_{n}^{*}=\arg\max_{l}\beta_{lk}\left|\forall n\neq k\right.\right\}\right)$ and $\mathrm{Unique}\left(T\right)$ returns the same values as those in the set T but with no repetitions. ${\hat{a}}_{lk}$ is the *l*th entry of ${\hat{a}}_{k}$, and ${\hat{a}}_{k}$ is the *k*th column of $\hat{A}$. $\eta_{\hat{l}k}$ is the channel gain of the *l*th AP and the *k*th user and is an RV with both large-scale fading coefficient and a small-scale fading coefficient. (*a*) is based on the consideration that when $p_{u}$ is high enough, $p_{u}\sigma_{{\hat{e}}_{\mathrm{lk}}}^{2}\approx \frac{1}{n}$. (*b*) occurs because $\frac{1}{{∥{\hat{a}}_{k}∥}^{2}}\approx \sum_{\hat{l}\in L_{k}}{\left|g_{\hat{l}k}\right|}^{2}$ [53]. Because the gain of small-scale fading ${\left|h_{lk}\right|}^{2}$ statistically follows the κ–μ shadowed distribution, the distribution of $\eta_{\hat{l}k}$ is obtained in Lemma 1 as follows.

**Lemma** **1.** *Based on a κ–μ shadowed fading model, the channel gain $\eta_{\hat{l}k}$ follows the Gamma distribution, which is*

(7) $$\eta_{\hat{l}k}∼\Gamma \left(K_{\hat{l}k},Q_{\hat{l}k}\right),$$

*where Kl^k=ml^kμlk1+κl^k2mll^k+μl^kκl^k2+2ml^kκl^k=μl^k1+κl^k21+μl^kκl^k2μl^kκl^k2mml^k+2κl^k, and $Q_{\hat{l}k}=\beta_{\hat{l}k}\theta_{\hat{l}k}=\frac{\beta_{\hat{l}k}\Omega_{\hat{l}k}}{K_{\hat{l}k}}=\frac{{\hat{\Omega }}_{\hat{l}k}}{K_{\hat{l}k}}$. $\beta_{\hat{l}k}\Omega_{\hat{l}k}$ is the mean value of $\eta_{\hat{l}k}$, as well as the mean value of the channel gain.*

**Proof.**  Please refer to Appendix A. □

Based on the κ–μ shadowed fading model, $\zeta_{k}=\sum_{\hat{l}\in L_{k}}\eta_{\hat{l}k}$ can be regarded to the sum of i.n.i.d. Gamma RVs. Then, the PDF, CDF and MGF of $\zeta_{k}$ are obtained from Theorem 1.

**Theorem** **1.** *Considering a κ–μ distribution with parameters ($A_{k},K_{k},{\hat{\Omega }}_{k}$), the PDF can be approximately obtained by a V-order generalized Puiseux series expansion process as follows.*

(8) $$f_{\zeta_{k}}\left(z;A_{k},K_{k},{\hat{\Omega }}_{k}\right)≅\sum_{v=1}^{V}c_{v}\frac{A_{k}D_{k}^{A_{k}K_{k}}}{{\left(\omega_{v}\Omega_{k}\right)}^{A_{k}K_{k}}\Gamma \left(K_{k}\right)}z^{A_{k}K_{k}-1}\exp\left(-{\left(D_{k}\frac{z}{\omega_{v}{\hat{\Omega }}_{k}}\right)}^{A_{k}}\right),$$

*where $A_{k}=A_{\hat{l}k}=1$, $K_{k}=\sum_{\hat{l}\in L_{k}}K_{\hat{l}k}$, $K_{k}=\sum_{\hat{l}\in L_{k}}K_{\hat{l}k}$ and Dk=ΓKk+11AkAkΓKk.*

*We can obtain approximate parameters $c_{v}$ and $\omega_{v}$ via the following formula:*

(9) ∑v=1Vcv=1,∑v=1Vcvωv=1,∑v=1Vcvωv2Ωk,22ΓKkΓKk+11AkAk2ΓKk+22AkAkΓKk=Eζk2,⋮⋮∑v=1Vcvωv2V−2Ωk,22V−2ΓKkΓKk+11AkAk2V−2ΓKk+2Mk−22Mk−2AkAkΓKk=Eζk2V−2,∑v=1VcvωvAkKkAkDkAkKkΩk,2AkKkΓKk=ALk,

*where $A_{L_{k}}=\left(\prod_{\hat{l}\in L_{k}}^{}\frac{AD_{\hat{l}k}^{AK_{\hat{l}k}}}{\Omega_{\hat{l}k,2}^{AK_{\hat{l}k}}\Gamma \left(K_{\hat{l}k}\right)}\right)\frac{\prod_{\hat{l}\in L_{k}}\Gamma \left(AK_{\hat{l}k}\right)}{\Gamma \left(A\sum_{\hat{l}\in L_{k}}K_{\hat{l}k}\right)}$ and*

$$E\left(\zeta_{k}^{n}\right)=\sum_{n_{1}=0}^{n}\sum_{n_{2}=0}^{n_{1}}\ldots \sum_{n_{\hat{L}-1}=0}^{n_{\hat{L}-2}}\left(\begin{array}{c} n\\ n_{1} \end{array}\right)\left(\begin{array}{c} n_{1}\\ n_{2} \end{array}\right)\ldots \left(\begin{array}{c} n_{\hat{L}-2}\\ n_{\hat{L}-1} \end{array}\right)E\left(X_{1}^{n-n_{1}}\right)E\left(X_{2}^{n_{1}-n_{2}}\right)\ldots E\left(X_{\hat{L}}^{n_{L-1}}\right).$$

*The CDF of $\zeta_{k}$ is given by*

(10) $$F_{\zeta_{k}}\left(z;A_{k},K_{k},{\hat{\Omega }}_{k}\right)≅\sum_{v=1}^{V}c_{v}\frac{\gamma \left(K_{k},{\left(D_{k}\frac{z}{\omega_{v}{\hat{\Omega }}_{k}}\right)}^{A_{k}}\right)}{\Gamma \left(K_{k}\right)},$$

*where $\gamma \left(\cdot ,\cdot \right)$ is the lower incomplete Gamma function and is expressed by*

(11) $$\gamma \left(s,x\right)=\int_{0}^{x}t^{s-1}\exp\left(-t\right)dt.$$

*The MGF of $\zeta_{k}$ is*

(12) Mζks=AkDkAkKkΩ^kAkKkΓKk∑v=1VcvωvAkKkq1/2pAkKk−1/22πp+qp+q22−1−sAkKkGp,qq,pDkωvΩ^kAkqpp−spqqΔp,1−AkKkΔq,0

*where $G_{p,q}^{q,p}\left(\cdot \right)$ is the Meijer G-function and $\frac{p}{q}=A_{k}=A_{n,k}=1$. According to $\gcd\left(p,q\right)=1$, both p and q can be 1.*

*Then, the MGF of $\zeta_{k}$ can be simplified to*

(13) $$M_{\zeta_{k}}\left(s\right)=\frac{A_{k}D_{k}^{A_{k}K_{k}}}{{\hat{\Omega }}_{k}^{A_{k}K_{k}}\Gamma \left(K_{k}\right)}\sum_{v=1}^{V}\frac{c_{v}}{\omega_{v}^{A_{k}K_{k}}}\frac{1}{{\left(-s\right)}^{A_{k}K_{k}}}G_{1,1}^{1,1}\left(\left.{\left(\frac{D_{k}}{\omega_{v}{\hat{\Omega }}_{k}}\right)}^{A_{k}}\frac{1}{-s}\right|\begin{array}{c} \Delta \left(1,1-A_{k}K_{k}\right)\\ \Delta \left(1,0\right) \end{array}\right).$$

**Proof.**  Please refer to Appendix B. □

Based on Equation (6) and Theorem 1, the statistical characteristics of the PPSNR in the CF mMIMO system are provided in the following corollary.

**Corollary** **1.** *Due to ${\hat{\gamma }}_{k}\approx {\hat{p}}_{u}\zeta_{k}$, the PDF of the PPSNR ${\hat{\gamma }}_{k}$ at the BBU is derived as*

(14) $$f_{{\hat{\gamma }}_{k}}\left(z;A_{k},K_{k},{\hat{p}}_{u}{\hat{\Omega }}_{k}\right)≅\sum_{v=1}^{V}c_{v}\frac{A_{k}D_{k}^{A_{k}K_{k}}}{{\left(\omega_{v}{\hat{p}}_{u}{\hat{\Omega }}_{k}\right)}^{A_{k}K_{k}}\Gamma \left(K_{k}\right)}z^{A_{k}K_{k}-1}\exp\left(-{\left(D_{k}\frac{z}{\omega_{v}{\hat{p}}_{u}{\hat{\Omega }}_{k}}\right)}^{A_{k}}\right).$$

*The CDF of ${\hat{\gamma }}_{k}$ is given by*

(15) $$F_{{\hat{\gamma }}_{k}}\left(z;A_{k},K_{k},{\hat{p}}_{u}{\hat{\Omega }}_{k}\right)≅\sum_{v=1}^{V}c_{v}\frac{\gamma \left(K_{k},{\left(D_{k}\frac{z}{\omega_{v}{\hat{p}}_{u}{\hat{\Omega }}_{k}}\right)}^{A_{k}}\right)}{\Gamma \left(K_{k}\right)}.$$

*The corresponding MGF of ${\hat{\gamma }}_{k}$ can be expressed as*

(16) $$\begin{array}{c} M_{\zeta_{k}}\left(s\right)\\ =\frac{A_{k}D_{k}^{A_{k}K_{k}}}{{\hat{\Omega }}_{k}^{A_{k}K_{k}}\Gamma \left(K_{k}\right)}\sum_{v=1}^{V}\frac{c_{v}}{\omega_{v}^{A_{k}K_{k}}}\frac{1}{{\left(-s\right)}^{A_{k}K_{k}}}G_{1,1}^{1,1}\left(\left.{\left(\frac{D_{k}}{\omega_{v}{\hat{\Omega }}_{k}}\right)}^{A_{k}}\frac{1}{-s}\right|\begin{array}{c} \Delta \left(1,1-A_{k}K_{k}\right)\\ \Delta \left(1,0\right) \end{array}\right) \end{array}.$$

## 3. Analysis of the Delay Performance

Because each packet is very short, both imperfect CSI and FBL channel coding cause transmission errors. The maximum achievable data rate is subject to transmission errors and an instantaneous signal-to-noise ratio (SNR), as described in Section 3.1. Due to transmission errors and dynamic data rates in the physical layer, the APs need to keep their packets in a buffer until successful completion, which leads to a random queuing delay, as discussed in Section 3.2. Finally, in Section 3.3, we derive the Mellin transform over the arrival and service processes and investigate the UB-QDVP.

### 3.1. Service Transmission in FBL Regime

Based on FBL information theory, the maximum achievable data rate (bit/CU) with an instantaneous SNR and a decoding error probability for the *k*th user can be obtained as follows. [22,33]

(17) $$\begin{array}{cc} r_{k}\left({\hat{\gamma }}_{k},\varepsilon_{k}^{d}\right) & =C\left({\hat{\gamma }}_{k}\right)-\sqrt{\frac{V\left({\hat{\gamma }}_{k}\right)}{\hat{n}}}Q^{-1}\left(\varepsilon_{k}^{d}\right)\\ \; & =\log_{2}^{\left(1+{\hat{\gamma }}_{k}\right)}-\log_{2}^{e}\sqrt{\frac{\left(1-\frac{1}{{\left(1+{\hat{\gamma }}_{k}\right)}^{2}}\right)}{\hat{n}}}Q^{-1}\left(\varepsilon_{k}^{d}\right)\\ \; & =\log_{2}^{\left(f_{r_{k}}\left({\hat{\gamma }}_{k},\varepsilon_{k}^{d}\right)\right)} \end{array},$$

where $f_{r_{k}}\left({\hat{\gamma }}_{k},\varepsilon_{k}^{d}\right)=\frac{1+{\hat{\gamma }}_{k}}{\exp\left(\sqrt{\frac{{\hat{\gamma }}_{k}^{2}+2{\hat{\gamma }}_{k}}{{\left(1+{\hat{\gamma }}_{k}\right)}^{2}\hat{n}}}Q^{-1}\left(\varepsilon_{k}^{d}\right)\right)}$, $\hat{n}$ is the number of CUs occupied by data transmission and $\varepsilon_{d}$ is the decoding error probability. Qx=12π∫x∞e−y2−y222dy is the tail distribution function of the standard normal distribution. $C\left({\hat{\gamma }}_{k}\right)=\log_{2}^{\left(1+{\hat{\gamma }}_{k}\right)}$ and $V\left({\hat{\gamma }}_{k}\right)=\left(1-\frac{1}{{\left(1+{\hat{\gamma }}_{k}\right)}^{2}}\right){\left(\log_{2}^{e}\right)}^{2}$ are the Shannon capacity and channel dispersion, respectively. Given a decoding error probability of $\varepsilon_{k}^{d}$, the achievable data rate $r_{k}\left({\hat{\gamma }}_{k},\varepsilon_{k}^{d}\right)$ is less than zero when the PPSNR ${\hat{\gamma }}_{k}$ is below a threshold $\gamma_{0}$. As a consequence, we redefine $f_{r_{k}}\left({\hat{\gamma }}_{k},\varepsilon_{k}^{d}\right)$ as $g_{r_{k}}\left({\hat{\gamma }}_{k},\varepsilon_{k}^{d}\right)$

(18) $$g_{r_{k}}\left({\hat{\gamma }}_{k},\varepsilon_{k}^{d}\right)=\left\{\begin{array}{c} f_{r_{k}}\left({\hat{\gamma }}_{k},\varepsilon_{k}^{d}\right),{\hat{\gamma }}_{k}>\gamma_{0}\\ 1,{\hat{\gamma }}_{k}\leq \gamma_{0} \end{array}\right..$$

Furthermore, the maximum achievable data rate can be simplified as follows:

(19) $$r_{k}\left({\hat{\gamma }}_{k},\varepsilon_{k}^{d}\right)=\left\{\begin{array}{c} \log_{2}^{f_{r_{k}}\left({\hat{\gamma }}_{k},\varepsilon_{k}^{d}\right)},{\hat{\gamma }}_{k}>\gamma_{0}\\ 0,{\hat{\gamma }}_{k}\leq \gamma_{0} \end{array}\right..$$

### 3.2. Queuing Model

For our queuing system analysis, we consider a usual statistical model such as that in [37], which is widely used in the SNC methodology. Then, the cumulative arrival, achievable service, and departure processes of user *k* during time interval [τ,t) are defined as follows.

(20) $$\left\{\begin{array}{c} A_{k}\left(\tau ,t\right)=\sum_{i=\tau }^{t-1}a_{k}\left(i\right),\\ R_{k}\left(\tau ,t\right)=\sum_{i=\tau }^{t-1}r_{k}\left(i\right),\\ U_{k}\left(\tau ,t\right)=\sum_{i=\tau }^{t-1}u_{k}\left(i\right). \end{array}\right.$$

The instantaneous traffic arrival $a_{k}\left(i\right)$ models the number of bits that arrive at the queue within a discrete time slot *i*. The increment of service process (service rate) $r_{k}\left(i\right)$ is independent of time and equal to the maximum achievable data rate $r_{k}\left({\hat{\gamma }}_{k},\varepsilon_{k}^{d}\right)$. The departure process $u_{k}\left(i\right)$ denotes the number of bits that arrive successfully at the destination and is subject to both the service process and the amount of data waiting in the queue. In addition, we assume that all queues are work-conserving first-come-first-served queues. Then, the queuing delay $w_{k}\left(t\right)$ of user *k* at time slot *t* is defined as the number of time slots required for all data that arrive before time *t* to depart from the transmit buffer and is expressed as

(21) $$w_{k}\left(t\right)=\inf\left\{u\geq 0:A_{k}\left(0,t\right)\leq U_{k}\left(0,t+u\right)\right\}.$$

### 3.3. SNC in the SNR Domain

A notable aspect of SNC is that it can obtain the delay distribution and the UB-QDVP based on statistical characterizations of the arrival and service processes in terms of their Mellin transforms. However, it is still difficult to capture the statistics of the random arrival and service processes in practical scenarios. To facilitate the analysis, we convert these processes from the bit domain through the exponential domain to the SNR domain. These cumulative processes in the SNR domain are denoted by calligraphic letters [54] as follows:

(22) $$\left\{\begin{array}{c} A_{k}\left(\tau ,t\right)=e^{A_{k}\left(\tau ,t\right)},\\ R_{k}\left(\tau ,t\right)=e^{R_{k}\left(\tau ,t\right)},\\ U_{k}\left(\tau ,t\right)=e^{U_{k}\left(\tau ,t\right)}. \end{array}\right.$$

$M_{X}\left(s\right)=E\left[X^{s-1}\right]$ is the Mellin transform of any nonnegative random variable X for a parameter s∈R [37]. Both $a_{k}\left(i\right)$ and $r_{k}\left(i\right)$ are independent of each other at different time slots, so we use two time-independent random variables $a_{k}$ and $r_{k}$ to denote the number of arrival and service bits at either time slot, respectively. Then, the Mellin transform over the accumulated arrival process is denoted by $M_{A_{k}}\left(s,\tau ,t\right)=M_{A_{k}\left(\tau ,t\right)}\left(s\right)=E\left({\left(e^{A_{k}\left(\tau ,t\right)}\right)}^{s-1}\right)={\left(M_{\varphi_{k}}\left(s\right)\right)}^{t-\tau }$, where $\varphi_{k}=e^{a_{k}}$. The Mellin transform over the accumulated achievable service process is $M_{R_{k}}\left(s,\tau ,t\right)=M_{R_{k}\left(\tau ,t\right)}\left(s\right)=E\left({\left(e^{R_{k}\left(\tau ,t\right)}\right)}^{s-1}\right)={\left(M_{\phi_{k}}\left(s\right)\right)}^{t-\tau }$, where $\phi_{k}=e^{r_{k}}$.

If the stability condition $M_{\varphi_{k}}\left(1+s\right)M_{\phi_{k}}\left(1-s\right)<1$ holds, denoting $t_{T}$ as a target delay (target number of time slots), the kernel function $K\left(s,t_{T}\right)$ is defined as [37]

(23) $$K\left(s,t_{T}\right)=\frac{M_{\phi_{k}}{\left(1-s\right)}^{t_{T}}}{1-M_{\varphi_{k}}\left(1+s\right)M_{\phi_{k}}\left(1-s\right)}.$$

Thus, the UB-QDVP denoted by $\varepsilon_{k}^{v,\mathrm{UB}}$ is [22,37]

(24) $$\varepsilon_{k}^{v,\mathrm{UB}}=\mathrm{Pr}\left\{w_{k}\left(t\right)>t_{T}\right\}\leq \underset{0<s<s_{0}}{\inf}K\left(s,t_{T}\right),$$

where $s_{0}=\sup\left\{s:M_{\varphi_{k}}\left(1+s\right)M_{\phi_{k}}\left(1-s\right)<1\right\}$.

#### 3.3.1. Mellin Transform over the Arrival Process in the SNR Domain

As shown in Equation (24), the UB-QDVP is related to the arrival process on the Mellin transform in the SNR domain. Consequently, deriving the Mellin transform of $a_{k}$ is essential for evaluating the UB-QDVP. We consider the traffic class of $\left(\delta \left(s\right),\lambda \left(s\right)\right)$-bounded arrivals, whose arrival processes characterized in the SNR domain for some s>0 on the Mellin transform are bounded by [37]

(25) $$M_{A_{k}}\left(s,\tau ,t\right)={\left(M_{\varphi_{k}}\left(s\right)\right)}^{t-\tau }\leq e^{\left(s-1)\cdot (\lambda (s-1)\cdot (t-\tau )+\delta (s-1)\right)}.$$

To simplify the notation, we consider the case where δ(s)=0 and λ(s)≡λ; namely, $\left(\delta \left(s\right),\lambda \left(s\right)\right)$ are independent of *s*, which is true for constant arrivals [54]. Thus,

(26) $$M_{\varphi_{k}}\left(1+s\right)\leq e^{\lambda s},$$

where λ is the constant number of arrival bits that arrive successfully at one slot, as well as the constant bit arrival rate.

#### 3.3.2. Mellin Transform over the Service Process in the SNR Domain

Equation (24) also shows that deriving the closed-form expression for the Mellin transform over the service process in the SNR domain at user *k* is a key component when analyzing the UB-QDVP, which motivates the following Theorem 2.

**Theorem** **2.** *The Mellin transform over the service process in the SNR domain of user k at one time slot is*

(27) $$M_{\phi_{k}}\left(1-s\right)\leq I_{k}\left(s,\varepsilon_{k}^{d}\right)+G_{k}\left(\gamma_{0},\varepsilon_{k}^{d}\right),$$

*where $I_{k}\left(s,\varepsilon_{k}^{d}\right)=\left(1-\varepsilon_{k}^{d}\right)M_{{\hat{\gamma }}_{k}}\left(-\frac{s}{\ln^{2}}\right)$ and $G_{k}\left(\gamma_{0},\varepsilon_{k}^{d}\right)=\varepsilon_{k}^{d}+\left(1-\varepsilon_{k}^{d}\right)F_{{\hat{\gamma }}_{k}}\left(\gamma_{0}\right)$. $\varepsilon_{k}^{d}$ is the decoding error probability. $\gamma_{0}$ is the upper bound of the PPSNR when the maximum achievable data rate of user k is 0.*

**Proof.**  Please refer to Appendix C. □

Combining and extending Theorem 2, Equations (24) and (26) yield the following corollary.

**Corollary** **2.** *The approximate UB-QDVP of the kth user is*

(28) $$\varepsilon_{k}^{v,\mathrm{UB}}≅\frac{{\left(I_{k}\left(s,\varepsilon_{k}^{d}\right)+G_{k}\left(\gamma_{0},\varepsilon_{k}^{d}\right)\right)}^{t_{T}}}{1-e^{\lambda s}\left(I_{k}\left(s,\varepsilon_{k}^{d}\right)+G_{k}\left(\gamma_{0},\varepsilon_{k}^{d}\right)\right)}.$$

**Proof.**  Please refer to Appendix D. □

## 4. Simulation Results and Analyses

We present numerical results to validate and evaluate the stochastic queuing behaviors and the effects of fading characteristics on performance in the proposed CF mMIMO system under the κ–μ shadowed fading model. Different from most studies [4,12,13,17,18], which explore the effects of the number of APs, the coverage radio, the number of potential users and other traditional system parameter configurations on performance in communication scenarios, we focus on fading characteristics. Throughout our simulations, we set such parameters constant. For large-scale fading $\beta_{lk}=u_{p}{\left(\frac{d_{k}}{d_{0}}\right)}^{-\alpha_{p}}$, $u_{p},\alpha_{p},d_{0}$ and $d_{lk}$ are the constant path loss across the minimum distance, the path loss exponent, the minimum distance and the distance between the *k*th user and the *l*th AP, respectively. The key simulation parameters are listed in Table 1. The overall reliability for the *k*th user can be represented by the overall error probability $\varepsilon_{k}$ [55], which is $\varepsilon_{k}\approx \varepsilon_{k}^{d}+\varepsilon_{k}^{v}$. Because $\varepsilon_{k}^{d}\to 0$, $\varepsilon_{k}$ can be expressed by $\varepsilon_{k}^{v,\mathrm{UB}}$.

**Figure 2:**
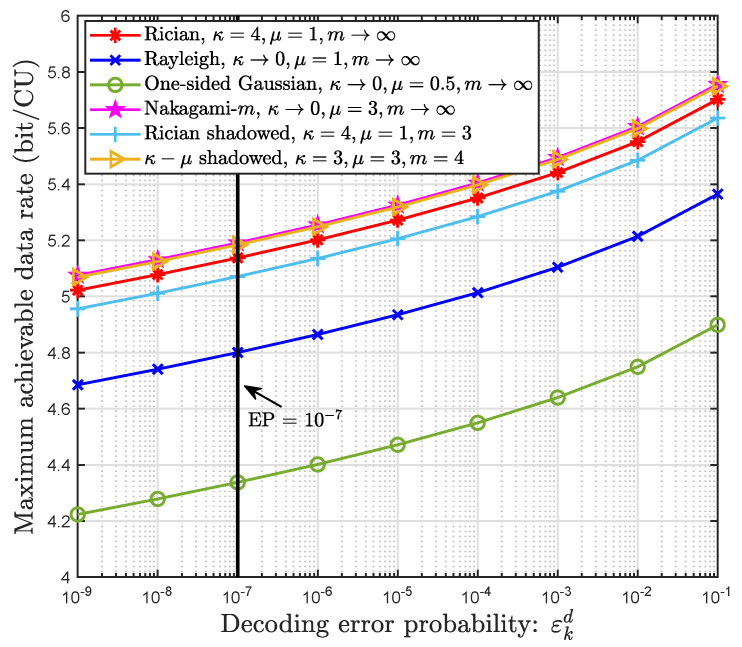
Relationship between the decoding error probability and the maximum achievable data rate.

First, we compared the maximum achievable data rates obtained under the same decoding error probability but different fading models in Figure 2, when the transmit power is 5 dBm and the distance from the user to the center of the coverage area is 200 m. It is obvious that the maximum achievable data rates corresponding to the same decoding error probability are inconsistent under different fading environments. This indicates that the effect of different fading environments cannot be ignored with respect to system performance. In particular, when designing a wireless communication system (e.g., the CF mMIMO system) for strict decoding error probability, it is crucial to design customized coding design and power control schemes by combining the fading characteristics of the wireless communication environment to adjust the service rate to meet the reliability requirements of NOMA-enabled URLLC QoS. Therefore, the κ–μ shadowed fading model accommodating different propagation conditions that are applied to design or analyze the performance of a wireless communication system is necessary and significant.

**Figure 3:**
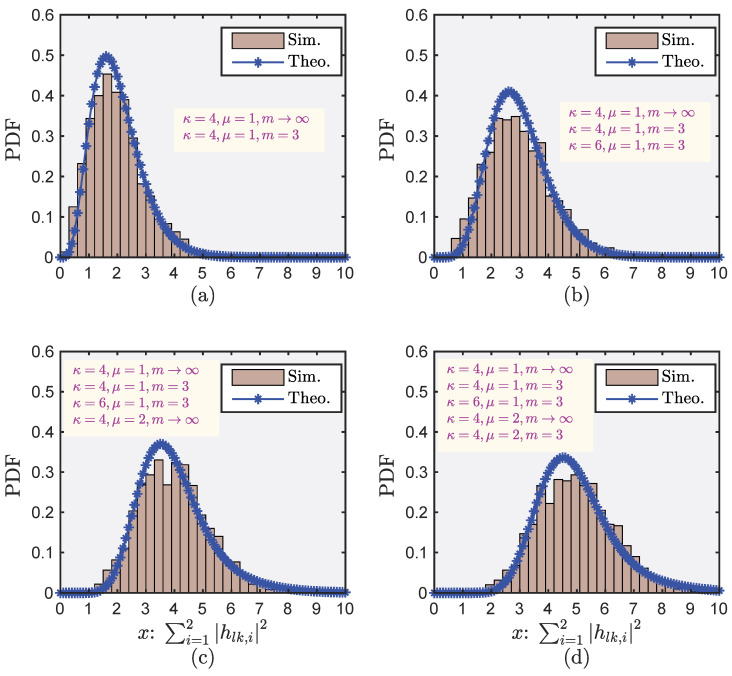
PDFs of the sum of i.n.i.d. κ–μ shadowed RVs. (**a**) Case 1; (**b**) Case 2; (**c**) Case 3; (**d**) Case 4.

Figure 3 shows the fitting ability of our derived theoretical tool (Theorem 1) for the PDFs of the sum of i.n.i.d. RVs. We used the Monte Carlo method to randomly generate six independent RVs with different distributions and sum some of them. We verify that the cylindrical diagram (obtained by a simulation using the Monte Carlo method for $10^{7}$ iterations) and the curve with an asterisk (obtained by numerical calculation (Equation (8)) have almost the same slope. This indicates that our proposed theoretical tools based on Theorem 1 can accurately characterize the statistical characteristics of the sum of multiple i.n.i.d. RVs. Furthermore, we summarize the second-order approximate parameters obtained under the different numbers of summed elements and different distributions listed in Table 2, which were obtained by using *fsolve* in MATLAB. From Figure 3, we also observe that the generalized Puiseux series expansion process converges very quickly, so it can effectively fit the PDF without complex approximate orders. All these properties lay the foundation that appropriately characterizing the statistical characteristics of the sum of multiple i.n.i.d. shadowed RVs is the key to unsolved problems. This again verifies the effectiveness and good analytical properties of our proposed theoretical tool (Theorem 1) in complex future communication environments.

**Figure 4:**
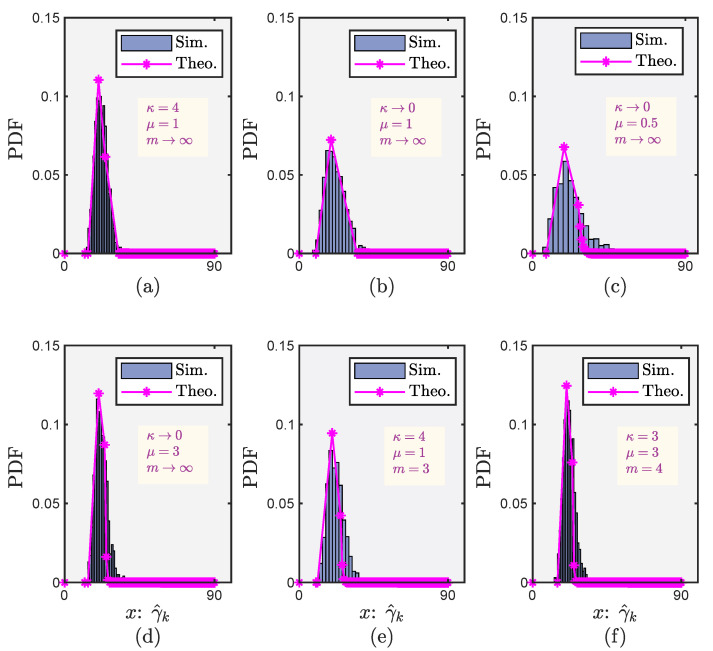
PDFs of the PPSNR for CF mMIMO system. (**a**) under the Rician fading channel; (**b**) under the Rayleigh fading channel; (**c**) under the One-sided fading channel; (**d**) under the Nakagami-*m* fading channel; (**e**) under the Rician shadwoed fading channel; (**f**) under the κ–μ shadowed fading channel.

For the proposed CF mMIMO system, Figure 4 depicts the PDFs of the PPSNR under different fading environments when the transmit power is 10 dBm and the distance from the user to the center of the coverage area is 200 m. We verify that the cylindrical diagram (obtained by simulation using Monte Carlo method for $10^{7}$ iterations) and the curve with asterisk (obtained by numerical calculation (Corollary 1)) have almost the same slope. This indicates that our proposed theoretical tools based on Corollary 1 have superior flexibility, mathematical tractability and practicality for wireless communication system design. Furthermore, these findings also reveal the κ–μ shadowed fading model based on our proposed theoretical tools, which form a reliable and mathematically tractable channel fading model can be extended to other common future 6G wireless communication systems such as CF mMIMO systems, enabling this model as a more applicable approach to complex 6G applications in the future.

**Figure 5:**
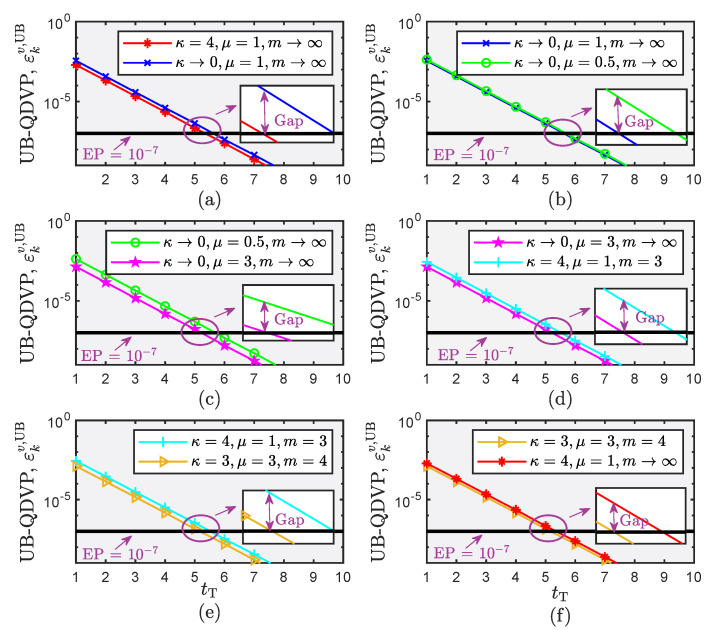
Performance comparison conducted for the CF mMIMO system under the κ–μ shadowed fading model. (**a**) Comparison between Rician and Rayleigh fading channel; (**b**) Comparison between Rayleigh and One-sided fading channel; (**c**) Comparison between One-sided and Nakagami-*m* fading channel; (**d**) Comparison between Nakagami-*m* and Rician shadowed fading channel; (**e**) Comparison between Rician shadowed and the κ–μ shadowed fading channel; (**f**) Comparison between the κ–μ shadowed and Rician fading channel.

Figure 5 plots the UB-QDVPs obtained with different target delays over a κ–μ shadowed fading model for the CF mMIMO system when the transmit power is 10 dBm and the distance from the user to the center of the coverage area is 200 m. The UB-QDVP decreases as the target delay increases. For the same target delay, the users have inconsistent UB-QDVPs under different fading environments. In Figure 5c, under Nakagami-*m* fading (κ→0,μ=3,m→∞) and a target delay of 0.05 ms, the UB-QDVP is $10^{-7}$. However, when the UB-QDVP is $10^{-7}$ under One-sided Gaussian fading (κ→0,μ=0.5,m→∞), the target delay exceeds 0.05 ms. For the performance evaluation, analysis and design of the system, it is necessary to consider the impacts of different fading environments on the system performance. Otherwise, the final result or conclusion obtained will differ greatly from reality, eventually leading to analysis results with less practical value and significance.

**Figure 6:**
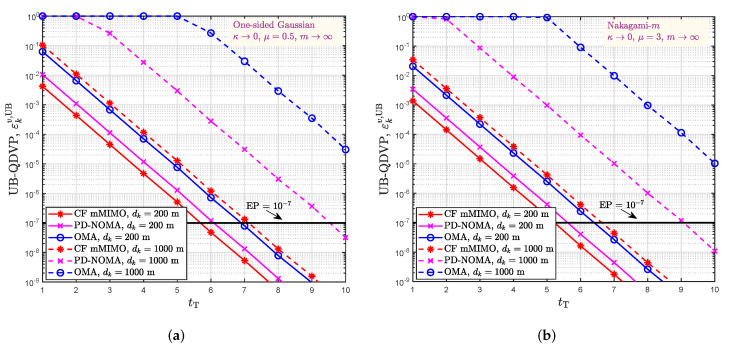
Comparison of the UB-QDVPs in the CF mMIMO, PD-NOMA and OMA systems. (**a**) Comparison of different communication systems under One-sided Gaussian fading; (**b**) Comparison of different communication systems under Nakagami-*m* fading.

Figure 6a compares the UB-QDVPs obtained under different distances from the user to the center of coverage area (200 m or 1000 m) in three communication systems under One-sided Gaussian fading (κ→0,μ=0.5,m→∞) when the transmit power is 10 dBm. These three communication systems are the CF mMIMO system we proposed, PD-NOMA and OMA systems equipped with 200 antennas in their base station. We observe that the UB-QDVP of the PD-NOMA system is lower than that of the OMA system, and the UB-QDVP of the CF mMIMO system is lower than that of the PD-NOMA system under the same target delay when the distance from the user to the center of the coverage area is 200 m. In detail, when the target delay is 0.06 ms and the distance from the user to the center of the coverage area is 200 m, the UB-QDVPs of the PD-NOMA and OMA systems exceed $10^{-7}$, and only the UB-QDVP of the CF mMIMO system is lower than $10^{-7}$. Under a target delay of 0.07 ms, the UB-QDVPs of the CF mMIMO system and the PD-NOMA system are below $10^{-7}$. However, under the same parameter settings, the UB-QDVP of the OMA system is still higher than $10^{-7}$. Specifically, we extended the distance from the user to the center of the coverage area to 1000 m and set the target delay to 0.08 ms. Only the CF mMIMO system can achieve a UB-QDVP lower than $10^{-7}$, and both the PD-NOMA and the OMA systems cannot. Figure 6b plots the UB-QDVP for a user at 200 m or 1000 m from the center of the coverage area under Nakagami-*m* fading (κ→0, μ=3, m→∞) for the CF mMIMO system, PD-NOMA system, and OMA system when the transmit power is 10 dBm. It is also clear from Figure 6b that when the user is 200 m from the center of the coverage area, the UB-QDVP of the CF mMIMO system is lower than that of the PD-NOMA system, which in turn is lower than that of the OMA system, for the same target delay. For example, when the target delay is 0.06 ms, the UB-QDVP is lower than $10^{-7}$ for the CF mMIMO system and PD-NOMA system, and higher than $10^{-7}$ for the OMA system when the user is 200 m away from the center of the coverage area. When the user is 1000 m away from the center of the coverage area and the target delay is 0.07 ms, only the UB-QDVP of the CF mMIMO system is lower than $10^{-7}$, and the UB-QDVPs of the PD-NOMA system and OMA system are higher than $10^{-7}$. Figure 6a,b confirm that the CF mMIMO system not only optimizes the reliability and delay performance, but also expands the coverage area for providing URLLC services in comparison with the PD-NOMA and OMA systems.

**Figure 7:**
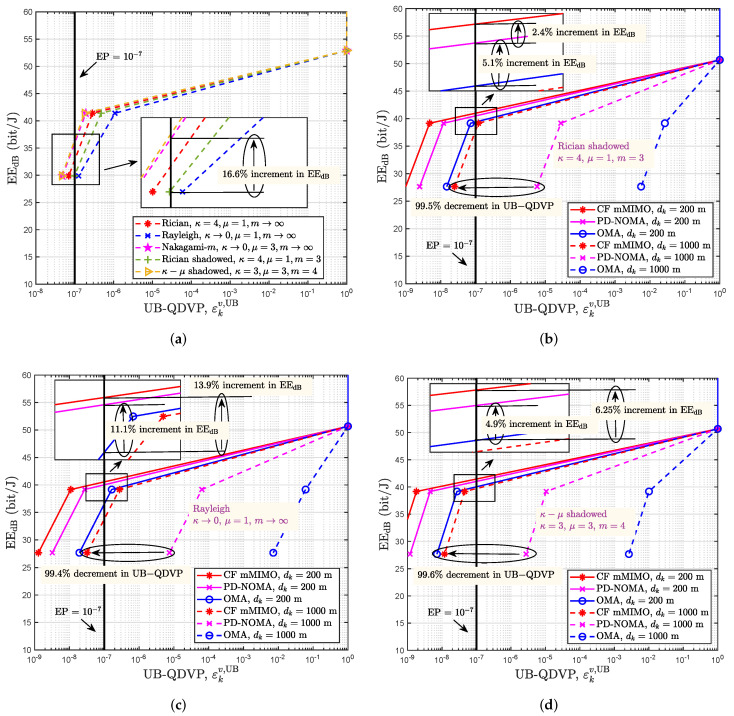
The UB-QDVP vs. $EE_{\mathrm{dB}}$ under the κ–μ shadowed fading model. (**a**) Comparison of different small-scale fading types; (**b**) Comparison of different communication systems under Rician shadowed fading; (**c**) Comparison of different communication systems under Rayleigh fading. (**d**) Comparison of different communication systems under κ–μ shadowed fading.

Figure 7a shows the relationship between $EE_{\mathrm{dB}}$ and the UB-QDVP under different fading environments in the proposed CF mMIMO communication system when the transmit power is 10 dBm, the target delay is 0.08 ms and the distance from the user to the center of the coverage area is 200 m. As seen from Figure 7a, as the UB-QDVP becomes tighter, $EE_{\mathrm{dB}}$ decreases. Under different fading environments, the $EE_{\mathrm{dB}}$ of the user for the same UB-QDVP is different, and a gap is observed. The $EE_{\mathrm{dB}}$ of the user under Nakagami-*m* fading (κ→0,μ=3,m→∞) for controlling the transmit power to meet the UB-QDVP requirement ($10^{-7}$) is improved by 16.6% in $EE_{\mathrm{dB}}$ over that of users under Rician shadowed fading (κ=4,μ=1,m=3) with the same UB-QDVP. Therefore, for delay-sensitive applications, performance analyses for indicators such as delay and reliability, power control and system design, the impacts of different small-scale fading characteristics on the performance of communication systems need to be considered. The accurate characterization of small-scale fading using the κ–μ shadowed fading model may become a necessity for future wireless communication performance analysis and system design.

Figure 7b–d compare the $EE_{\mathrm{dB}}$ and UB-QDVP values obtained under different distances from the user to the center of the coverage area (200 m or 1000 m) in three communications systems under Rician shadowed fading (κ=4,μ=1,m=3), Rayleigh fading (κ→0,μ=1,m→∞) and κ–μ shadowed fading (κ=3,μ=3,m=4) when the transmit power is 10 dBm, respectively. These three communication systems are the proposed CF mMIMO system, PD-NOMA and OMA systems equipped with 200 antennas in their base station. In Figure 7b, we can see that when the target delay is 0.1 ms and the distance to the center of the coverage area is 200 m, the user achieves $\varepsilon_{k}^{v,\mathrm{UB}}=10^{-7}$ with $EE_{\mathrm{dB}}<40$ bit/J in the OMA system. Under the same conditions, the user in the PD-NOMA system can arrive at the same UB-QDVP with $EE_{\mathrm{dB}}>40$ bit/J, which is an $EE_{\mathrm{dB}}$ improvement of 5.1% over that of the OMA system. Furthermore, the user in the CF mMIMO system achieves a 2.4% increment in $EE_{\mathrm{dB}}$ compared with that of the PD-NOMA system. When $EE_{\mathrm{dB}}=27.65$ bit/J, the target delay is 0.1 ms and the distance to the center of the coverage area expands to 1000 m, the UB-QDVPs of the user in both PD-NOMA and OMA systems are greater than $10^{-7}$. However, the UB-QDVP of the user in the CF mMIMO system is less than $10^{-7}$ and is 97.5% lower than that of the PD-NOMA system. In Figure 7c, when the target delay is 0.1 ms and the distance to the center of the coverage area is 200 m, the user achieves $\varepsilon_{k}^{v,\mathrm{UB}}=10^{-7}$ with $EE_{\mathrm{dB}}<40$ bit/J in the OMA system. Under the same conditions, the user in the PD-NOMA system can arrive at the same UB-QDVP with $EE_{\mathrm{dB}}=40$ bit/J, which is an $EE_{\mathrm{dB}}$ improvement of 11.1% over that of the OMA system. Furthermore, the user in the CF mMIMO system achieves a 13.9% increment in $EE_{\mathrm{dB}}$ compared with that of the OMA system. When $EE_{\mathrm{dB}}=27.65$ bit/J, the target delay is 0.1 ms and the distance to the center of the coverage area expands to 1000 m, the UB-QDVPs of the user in both PD-NOMA and OMA systems are greater than $10^{-7}$. However, the UB-QDVP of the user in the CF mMIMO system is less than $10^{-7}$ and is 99.4% lower than that of the PD-NOMA system. In Figure 7d, when the target delay is 0.1 ms and the distance to the center of the coverage area is 200 m, the user achieves $\varepsilon_{k}^{v,\mathrm{UB}}=10^{-7}$ with $EE_{\mathrm{dB}}=40$ bit/J in the OMA system. Under the same conditions, the user in the PD-NOMA system can arrive at the same UB-QDVP with $EE_{\mathrm{dB}}>40$ bit/J, which is an $EE_{\mathrm{dB}}$ improvement of 4.9% over that of the OMA system. Furthermore, the user in the CF mMIMO system achieves a 6.25% increment in $EE_{\mathrm{dB}}$ compared with that of the OMA system. When $EE_{\mathrm{dB}}=27.65$ bit/J, the target delay is 0.1 ms and the distance to the center of the coverage area expands to 1000 m, the UB-QDVPs of the user in both PD-NOMA and OMA systems are greater than $10^{-7}$. However, the UB-QDVP of the user in the CF mMIMO system is less than $10^{-7}$ and is 99.6% lower than that of the PD-NOMA system. Hence, the CF mMIMO approach is a prospective technology that can improve EE while ensuring that the target delay and reliability meet the URLLC QoS requirements. At the same time, it can greatly decrease the UB-QDVP and improve user reliability, thereby ensuring ultrareliable communication for all users covered.

## 5. Conclusions

In this paper, we developed an analytical model to characterize stochastic queuing behaviors and the effects of fading characteristics on performance in the proposed CF mMIMO system under the κ–μ shadowed fading model. We derived approximate closed-form expressions for the PDF, CDF and MGF of the sum of i.n.i.d. κ–μ shadowed RVs and improved their mathematical tractability. Then, based on the κ–μ shadowed fading model, we derived approximate closed-form expressions for the PDF, CDF and MGF of the PPSNR after the zero-forcing detector in the proposed CF mMIMO system. The κ–μ shadowed fading model can be extended to 6G wireless communication systems, and we verified the reasonableness and feasibility of its superior theoretical analysis performance with simulations and theoretical comparisons. In particular, we applied FBL information theory and SNC to model and characterize the arrival, service and departure processes, and we derived a closed-form expression for the UB-QDVP to analyze the effectiveness and reliability of the CF mMIMO system under the κ–μ shadowed fading model. Furthermore, by applying the theoretical analysis above, we also conducted a set of simulations to validate and evaluate the UB-QDVP gaps under different systems (the CF mMIMO, PD-NOMA and OMA systems) and fading environments and the relationships between the UB-QDVPs and EE under different fading environments. Making full use of the characteristics of the κ–μ shadowed fading model can improve the system EE, reduce the error probability and better satisfy the QoS requirements of 6G URLLC. Applying the κ–μ shadowed fading model to accurately describe small-scale fading and to model and analyze unmanned aerial vehicles, underwater acoustics and other wireless communication systems in the future is a promising direction.

## Figures and Tables

**Table 1:** Parameters Values for Numerical Results.

Parameter	Value
Farthest coverage radius (m)	1000
Number of potential users	2000
Activation rate of potential users	5%
Number of APs	200
Noise power spectral density (dBm/Hz)	−106
Path loss exponent, $\alpha_{p}$	3
Minimum distance, $d_{0}$ (m)	10
Constant path loss, $u_{p}$ (dB)	−10
Bandwidth, *B* (MHz)	20
Carrier frequency (GHz)	3.5
Arrival bit per slot, λ (bit/slot)	50
Length of time slot, $t_{f}$ (ms)	0.01
Length of pilots, *n*	Max(K,0.2N) ^1^
Target delay (target number of time slots), $t_{T}$	[1,10]
Decoding error probability, $\varepsilon_{k}^{d}$	[$10^{-9},10^{-8}$]

^1^ This is the fixed ratio pilot allocation method [56].

**Table 2:** The second-order approximate parameters in different cases shown in Figure 3.

	$c_{1}$	$c_{2}$	$\omega_{1}$	$\omega_{2}$
Case 1:				
κ=4,μ=1,m→∞ κ=4,μ=1,m=3	0.9998	0.0002	0.9976	16.6538
Case 2:				
κ=4,μ=1,m→∞ κ=4,μ=1,m=3 κ=6,μ=1,m=3	0.9985	0.0015	0.9923	5.9651
Case 3:				
κ=4,μ=1,m→∞ κ=4,μ=1,m=3 κ=6,μ=1,m=3 κ=4,μ=2,m→∞	0.8605	0.1395	0.9355	1.3979
Case 4:				
κ=4,μ=1,m→∞ κ=4,μ=1,m=3 κ=6,μ=1,m=3 κ=4,μ=2,m→∞ κ=4,μ=2,m=3	0.8902	0.1098	0.9486	1.4166

## Data Availability

Not applicable.

## Abbreviations

The following abbreviations are used in this manuscript.

URLLC	Ultrareliable and low-latency communications
CF mMIMO	Cell-free massive multiple-input multiple-output
NOMA	Nonorthogonal multiple access
UB-QDVP	Upper bound of queuing delay violation probability
QoS	Quality-of-service
EE	Energy efficiency
OMA	Orthogonal multiple access
CU	Channel use
PD-NOMA	Power-domain NOMA
AP	Access point
FBL	Finite blocklength
SNC	Stochastic network calculus
CSI	Channel state information
PDF	Probability density function
CDF	Cumulative distribution function
SINR	Signal-to-noise-plus-interference ratio
MGF	Moment-generating function
i.n.i.d.	Independent and nonidentically distributed
BBU	Baseband unit
i.i.d.	Independent and identically distributed
AWGN	Additive white Gaussian noise

## Appendix A. Proof of Lemma 1

According to ${\left|h_{lk}\right|}^{2}∼\Gamma \left(K_{lk},\theta_{lk}\right)$, $\eta_{\hat{l}k}=\beta_{\hat{l}k}{\left|h_{\hat{l}k}\right|}^{2}$ and the characteristics of the Gamma distribution, we can obtain $\eta_{\hat{l}k}∼\Gamma \left(K_{\hat{l}k},\beta_{\hat{l}k}\theta_{\hat{l}k}\right)$; thus, $\eta_{\hat{l}k}∼\Gamma \left(K_{\hat{l}k},Q_{\hat{l}k}\right)$, where Kl^k=mμ1+κ2m+μκ2+2mκ=μ1+κ21+μκ2μκ2mm+2κ and $Q_{\hat{l}k}=\beta_{\hat{l}k}\theta_{\hat{l}k}=\frac{\beta_{\hat{l}k}\Omega_{\hat{l}k}}{K_{\hat{l}k}}=\frac{{\hat{\Omega }}_{\hat{l}k}}{K_{\hat{l}k}}$. Lemma 1 is proven.

## Appendix B. Proof of Theorem 1

In reference [57], the authors derived the PDF of the sum of i.n.i.d. generalized Gamma RVs. Therefore, we transform the PDF of $\eta_{\hat{l}k}$ into the PDF of generalized Gamma RVs, and its expression is as follows.

(A1) $$\begin{array}{cc} f_{\eta_{\hat{l}k}}\left(x;\alpha ,\kappa ,\mu ,m,{\hat{\Omega }}_{\hat{l}k}\right) & =f_{\zeta_{m,k}}\left(x;A_{\hat{l}k},K_{\hat{l}k},{\hat{\Omega }}_{\hat{l}k}\right)\\ \; & =\frac{A_{\hat{l}k}D_{\hat{l}k}^{A_{\hat{l}k}K_{\hat{l}k}}}{{\hat{\Omega }}_{\hat{l}k}^{A_{\hat{l}k}K_{\hat{l}k}}\Gamma \left(K_{\hat{l}k}\right)}x^{A_{\hat{l}k}K_{\hat{l}k}-1}\exp\left(-{\left(D_{\hat{l}k}\frac{x}{{\hat{\Omega }}_{\hat{l}k}}\right)}^{A_{\hat{l}k}}\right) \end{array}$$

where Dl^k=ΓKl^k+11Al^kAl^kΓKl^k. For $\hat{l}\in L_{k}$ and $k\in \left[1,K\right]$, $A_{\hat{l}k}=1$ can be obtained.

Therefore, when $\hat{l}\in L_{k},k\in \left[1,K\right]$ and $A_{\hat{l}k}$ is constant, the sum of $\hat{L}$ i.n.i.d. generalized Gamma RVs can be approximated by the generalized Puiseux series expansion. The approximation for the PDF of the sum of *L* i.n.i.d. κ–μ shadowed RVs *z* is given by

(A2) $$\begin{array}{cc} f_{Z}\left(z\right) & =\prod_{i=1}^{L}\frac{AD_{i}^{AK_{i}}}{\Omega_{i,2}^{AK_{i}}\Gamma \left(K_{i}\right)}z^{A\sum_{i=1}^{L}K_{i}-1}\frac{\prod_{i=1}^{L}\Gamma \left(AK_{i}\right)}{\Gamma \left(A\sum_{i=1}^{L}K_{i}\right)}I\left(z;u_{1},u_{2},\ldots ,u_{L-1}\right)\\ = & A_{L}z^{A\sum_{i=1}^{L}K_{i}-1}I\left(z;u_{1},u_{2},\ldots ,u_{L-1}\right) \end{array}$$

where

(A3) $$A_{L}=\left(\prod_{i=1}^{L}\frac{AD_{i}^{AK_{i}}}{\Omega_{i,2}^{AK_{i}}\Gamma \left(K_{i}\right)}\right)\frac{\prod_{i=1}^{L}\Gamma \left(AK_{i}\right)}{\Gamma \left(A\sum_{i=1}^{L}K_{i}\right)}$$

(A4) $$\begin{array}{c} I\left(z;u_{1},u_{2},\ldots ,u_{L-1}\right)\\ =\int_{0}^{1}\int_{0}^{1}\ldots \int_{0}^{1}\prod_{k=1}^{L-1}\frac{u_{k}^{A\sum_{j=1}^{k}K_{j}-1}{\left(1-u_{k}\right)}^{AK_{k+1}-1}}{B\left(A\sum_{i=1}^{k}K_{k},AK_{k+1}\right)}\Xi^{\psi \left(u_{1},u_{2},\ldots u_{L-1}\right)}du_{1}du_{2}\ldots du_{L-1} \end{array}$$

(A5) $$\Xi =\exp\left(-{\left(z\right)}^{A}\right)$$

(A6) $$\begin{array}{c} \psi \left(u_{1},u_{2},\ldots u_{L-1}\right)\\ =\left(\left(\begin{array}{c} \ldots \left(\left({\left(D_{1}\frac{u_{1}}{\Omega_{1,2}}\right)}^{A}+{\left(D_{2}\frac{\left(1-u_{1}\right)}{\Omega_{2,2}}\right)}^{A}\right)u_{2}^{A}+{\left(D_{3}\frac{\left(1-u_{2}\right)}{\Omega_{3,2}}\right)}^{A}\right)u_{3}^{A}\\ +{\left(D_{4}\frac{\left(1-u_{3}\right)}{\Omega_{4,2}}\right)}^{A}\ldots \end{array}\right)u_{L-1}^{A}+{\left(D_{L}\frac{\left(1-u_{L-1}\right)}{\Omega_{L,2}}\right)}^{A}\right) \end{array}$$

On the basis of the generalized Puiseux series expansion process, $I\left(z;u_{1},u_{2},\ldots ,u_{L-1}\right)$ can be approximated by the truncation order of any integer *V*, namely,

(A7) $$I\left(z;u_{1},u_{2},\ldots ,u_{L-1}\right)=\sum_{v=1}^{V}{c^{\prime }}_{v}\Xi^{\sigma_{v}}$$

When V→∞, the series can accurately approach $I\left(z;u_{1},u_{2},\ldots ,u_{L-1}\right)$. Moreover, the series expansion converges very quickly. Equation (A2) can be expressed as

(A8) $$\begin{array}{cc} f_{Z}\left(z\right) & ≅\sum_{v=1}^{V}{c^{\prime }}_{v}A_{L}z^{A\sum_{i=1}^{L}K_{i}-1}\Xi^{\sigma_{v}}\\ \; & ≅\sum_{v=1}^{V}{c^{\prime }}_{v}A_{L}z^{A\sum_{i=1}^{L}K_{i}-1}\exp\left(-\sigma_{v}z^{A}\right) \end{array}$$

To simplify the derivation, we transform the content of the cumulative sum into the PDF of the generalized Gamma distribution as follows.

(A9) $$\begin{array}{cc} f_{Z}\left(z\right) & ≅\sum_{v=1}^{V}{c^{\prime }}_{v}A_{L}z^{A\sum_{i=1}^{L}K_{i}-1}\exp\left(-\sigma_{v}z^{A}\right)\\ \; & ≅\sum_{v=1}^{V}{c^{\prime }}_{v}\left(\prod_{i=1}^{L}\frac{AD_{i}^{AK_{i}}}{\Omega_{i,2}^{AK_{i}}\Gamma \left(K_{i}\right)}\right)\frac{\prod_{i=1}^{L}\Gamma \left(AK_{i}\right)}{\Gamma \left(A\sum_{i=1}^{L}K_{i}\right)}z^{A\sum_{i=1}^{L}K_{i}-1}\exp\left(-\sigma_{v}z^{A}\right)\\ \; & ≅\sum_{v=1}^{V}{c^{\prime }}_{v}\left(\prod_{i=1}^{L}\frac{AD_{i}^{AK_{i}}}{\Omega_{i,2}^{AK_{i}}\Gamma \left(K_{i}\right)}\right)\frac{\prod_{i=1}^{L}\Gamma \left(AK_{i}\right)}{\Gamma \left(A\sum_{i=1}^{L}K_{i}\right)}\frac{{\left(\omega_{v}\Omega_{z,2}\right)}^{A_{z}K_{z}}\Gamma \left(K_{z}\right)}{A_{z}D_{z}^{A_{z}K_{z}}}\frac{A_{z}D_{z}^{A_{z}K_{z}}}{{\left(\omega_{v}\Omega_{z,2}\right)}^{A_{z}K_{z}}\Gamma \left(K_{z}\right)}z^{AK_{z}-1}\exp\left(-{\left(\frac{D_{z}}{\omega_{v}\Omega_{z,2}}z\right)}^{A}\right)\\ \; & ≅\sum_{v=1}^{V}c_{v}\frac{A_{z}D_{z}^{A_{z}K_{z}}}{{\left(\omega_{v}\Omega_{z,2}\right)}^{A_{z}K_{z}}\Gamma \left(K_{z}\right)}z^{A_{z}K_{z}-1}\exp\left(-{\left(D_{z}\frac{z}{\omega_{v}\Omega_{z,2}}\right)}^{A}\right) \end{array}$$

Additionally, according to the nature of the Gamma distribution, the corresponding CDF of the approximation can be estimated as

(A10) $$F_{Z}\left(z\right)≅\sum_{v=1}^{V}c_{v}\frac{\gamma \left(K,{\left(D_{z}\frac{z}{\omega_{v}\Omega_{z,2}}\right)}^{A}\right)}{\Gamma \left(K_{z}\right)}$$

The coefficients $c_{m}$ and $\omega_{m}$ can be obtained by using the moment-matching method [57]. Using this approach, the moments of the approximate PDF are equal to the exact moments. For this purpose, the following equations are formulated, in which the approximate moments appear on the left and the exact moments appear on the right.

(A11) ∑v=1Vcv=1,∑v=1VcvωvΩz,2=EZ=EX1+X2+…+XL=∑i=1LEXi∑v=1Vcvωv2Ωz,22ΓKzΓKz+11AzAz2ΓKz+22AzAzΓKz=EZ2⋮⋮∑v=1Vcvωv2V−2Ωz,22V−2ΓKzΓKz+11AzAz2V−2ΓKz+2Mz−22Mz−2AzAzΓKz=EZ2V−2∑v=1VcvωvAzKzAzDzAzKzΩz,2AzKzΓKz=∑v=1Vc′vAL=AL

Theorem 1 is proven.

## Appendix C. Proof of Theorem 2

The Mellin transform over the service process of user *k* at one time slot is

(A12) $$\begin{array}{cc} M_{\phi_{k}}\left(1-s\right) & =E\left(e^{\left(1-s-1\right)r_{k}\left({\hat{\gamma }}_{k},\varepsilon_{k}^{d}\right)}\right)\\ \; & =\int_{0}^{\infty }\left(\varepsilon_{k}^{d}+\left(1-\varepsilon_{k}^{d}\right)e^{-sr_{k}\left({\hat{\gamma }}_{k},\varepsilon_{k}^{d}\right)}\right)f_{\gamma }\left(x\right)dx\\ \; & =\varepsilon_{k}^{d}+\left(1-\varepsilon_{k}^{d}\right)\int_{\gamma_{0}}^{\infty }{\left(g_{r_{k}}\left({\hat{\gamma }}_{k},\varepsilon_{k}^{d}\right)\right)}^{-\frac{s}{\ln^{2}}}f_{{\hat{\gamma }}_{k}}\left(x\right)dx+\left(1-\varepsilon_{k}^{d}\right)\int_{0}^{\gamma_{0}}{\left(g_{r_{k}}\left({\hat{\gamma }}_{k},\varepsilon_{k}^{d}\right)\right)}^{-\frac{s}{\ln^{2}}}f_{{\hat{\gamma }}_{k}}\left(x\right)dx\\ \; & \overset{\left(c\right)}{=}\varepsilon_{k}^{d}+\left(1-\varepsilon_{k}^{d}\right)\int_{\gamma_{0}}^{\infty }{\left(f_{r_{k}}\left({\hat{\gamma }}_{k},\varepsilon_{k}^{d}\right)\right)}^{-\frac{s}{\ln^{2}}}f_{{\hat{\gamma }}_{k}}\left(x\right)dx+\left(1-\varepsilon_{k}^{d}\right)\int_{0}^{\gamma_{0}}f_{{\hat{\gamma }}_{k}}\left(x\right)dx\\ \; & =\varepsilon_{k}^{d}+\left(1-\varepsilon_{k}^{d}\right)\int_{\gamma_{0}}^{\infty }{\left(f_{r_{k}}\left({\hat{\gamma }}_{k},\varepsilon_{k}^{d}\right)\right)}^{-\frac{s}{\ln^{2}}}f_{{\hat{\gamma }}_{k}}\left(x\right)dx+\left(1-\varepsilon_{k}^{d}\right)\left(F_{{\hat{\gamma }}_{k}}\left(\gamma_{0}\right)-F_{{\hat{\gamma }}_{k}}\left(0\right)\right)\\ \; & ⩽\varepsilon_{k}^{d}+\left(1-\varepsilon_{k}^{d}\right)\int_{0}^{\infty }{\left(f_{r_{k}}\left({\hat{\gamma }}_{k},\varepsilon_{k}^{d}\right)\right)}^{-\frac{s}{\ln^{2}}}f_{{\hat{\gamma }}_{k}}\left(x\right)dx+\left(1-\varepsilon_{k}^{d}\right)F_{{\hat{\gamma }}_{k}}\left(\gamma_{0}\right)\\ \; & \;=\;\varepsilon_{k}^{d}+\left(1-\varepsilon_{k}^{d}\right)\int_{0}^{\infty }{\left(\frac{1+{\hat{\gamma }}_{k}}{\exp\left(\sqrt{\frac{{\hat{\gamma }}_{k}^{2}+2{\hat{\gamma }}_{k}}{{\left(1+{\hat{\gamma }}_{k}\right)}^{2}\hat{n}}}Q^{-1}\left(\varepsilon_{k}^{d}\right)\right)}\right)}^{-\frac{s}{\ln^{2}}}f_{{\hat{\gamma }}_{k}}\left(x\right)dx+\left(1-\varepsilon_{k}^{d}\right)F_{{\hat{\gamma }}_{k}}\left(\gamma_{0}\right). \end{array}$$

When ${\hat{\gamma }}_{k}⩽\gamma_{0}$, $g_{r_{k}}\left({\hat{\gamma }}_{k},\varepsilon_{k}^{d}\right)=\left\{\begin{array}{c} f_{r_{k}}\left({\hat{\gamma }}_{k},\varepsilon_{k}^{d}\right),{\hat{\gamma }}_{k}>\gamma_{0}\\ 1,{\hat{\gamma }}_{k}⩽\gamma_{0} \end{array}\right.$, (*c*) is established.

When ${\hat{\gamma }}_{k}\gg 1$, $V\left({\hat{\gamma }}_{k}\right)=\frac{{\hat{\gamma }}_{k}^{2}+2{\hat{\gamma }}_{k}}{{\left(1+{\hat{\gamma }}_{k}\right)}^{2}}\to 1$, we can obtain $\sqrt{\frac{{\hat{\gamma }}_{k}^{2}+2{\hat{\gamma }}_{k}}{{\left(1+{\hat{\gamma }}_{k}\right)}^{2}\hat{n}}}Q^{-1}\left(\varepsilon \right)\to 0$ and $\frac{1+{\hat{\gamma }}_{k}}{\exp\left(\sqrt{\frac{{\hat{\gamma }}_{k}^{2}+2{\hat{\gamma }}_{k}}{{\left(1+{\hat{\gamma }}_{k}\right)}^{2}\hat{n}}}Q^{-1}\left(\varepsilon \right)\right)}\to {\hat{\gamma }}_{k}$ [22].

The Mellin transform over the service process in the SNR domain of the *k*th user at one time slot can be rewritten as

(A13) $$\begin{array}{cc} M_{\phi_{k}}\left(1-s\right) & \leq \varepsilon_{k}^{d}+\left(1-\varepsilon_{k}^{d}\right)\int_{0}^{\infty }{\left(\frac{1+{\hat{\gamma }}_{k}}{\exp\left(\sqrt{\frac{{\hat{\gamma }}_{k}^{2}+2{\hat{\gamma }}_{k}}{{\left(1+{\hat{\gamma }}_{k}\right)}^{2}\hat{n}}}Q^{-1}\left(\varepsilon \right)\right)}\right)}^{-\frac{s}{\ln^{2}}}f_{{\hat{\gamma }}_{k}}\left(x\right)dx+\left(1-\varepsilon_{k}^{d}\right)F_{{\hat{\gamma }}_{k}}\left(\gamma_{0}\right)\\ \; & \overset{\left(d\right)}{\approx }\varepsilon_{k}^{d}+\left(1-\varepsilon_{k}^{d}\right)\int_{0}^{\infty }{\hat{\gamma }}_{k}^{-\frac{s}{\ln^{2}}}f_{{\hat{\gamma }}_{k}}\left(x\right)dx+\left(1-\varepsilon_{k}^{d}\right)F_{{\hat{\gamma }}_{k}}\left(\gamma_{0}\right)\\ \; & =\varepsilon_{k}^{d}+\left(1-\varepsilon_{k}^{d}\right)\int_{0}^{\infty }e^{-\frac{s}{\ln^{2}}\ln^{{\hat{\gamma }}_{k}}}f_{{\hat{\gamma }}_{k}}\left(x\right)dx+\left(1-\varepsilon_{k}^{d}\right)F_{{\hat{\gamma }}_{k}}\left(\gamma_{0}\right)\\ \; & \overset{\left(e\right)}{\approx }\varepsilon_{k}^{d}+\left(1-\varepsilon_{k}^{d}\right)\int_{0}^{\infty }e^{-\frac{s}{\ln^{2}}\left({\hat{\gamma }}_{k}-1\right)}f_{{\hat{\gamma }}_{k}}\left(x\right)dx+\left(1-\varepsilon_{k}^{d}\right)F_{{\hat{\gamma }}_{k}}\left(\gamma_{0}\right)\\ \; & \overset{\left(f\right)}{\approx }\varepsilon_{k}^{d}+\left(1-\varepsilon_{k}^{d}\right)M_{\gamma_{k}}\left(-\frac{s}{\ln^{2}}\right)+\left(1-\varepsilon_{k}^{d}\right)F_{{\hat{\gamma }}_{k}}\left(\gamma_{0}\right)\\ \; & =I_{k}\left(s,\varepsilon_{k}^{d}\right)+G_{k}\left(\gamma_{0},\varepsilon_{k}^{d}\right) \end{array}$$

where $I_{k}\left(s,\varepsilon_{k}^{d}\right)=\left(1-\varepsilon_{k}^{d}\right)M_{{\hat{\gamma }}_{k}}\left(-\frac{s}{\ln^{2}}\right)$, $G_{k}\left(\gamma_{0},\varepsilon_{k}^{d}\right)=\varepsilon_{k}^{d}+\left(1-\varepsilon_{k}^{d}\right)F_{{\hat{\gamma }}_{k}}\left(\gamma_{0}\right)$. (*d*) and (*f*) are obtained from ${\hat{\gamma }}_{k}\gg 1$. (*e*) is approximately obtained in terms of the first-order expansion of the Taylor formula. Theorem 2 is proven.

## Appendix D. Proof of Corollary 2

The approximate UB-QDVP of the *k*th user is

(A14) $$\begin{array}{cc} \varepsilon_{k}^{v,\mathrm{UB}} & =\mathrm{Pr}\left\{w_{k}\left(t\right)>t_{T}\right\}\\ \; & \leq \frac{M_{\phi_{k}}{\left(1-s\right)}^{t_{T}}}{1-M_{\varphi_{k}}\left(1+s\right)M_{\phi_{k}}\left(1-s\right)}\\ \; & \leq \frac{M_{\phi_{k}}{\left(1-s\right)}^{t_{T}}}{1-e^{\lambda s}M_{\phi_{k}}\left(1-s\right)}\\ \; & \leq \frac{{\left(I_{k}\left(s,\varepsilon_{k}^{d}\right)+G_{k}\left(\gamma_{0},\varepsilon_{k}^{d}\right)\right)}^{t_{T}}}{1-e^{\lambda s}\left(I_{k}\left(s,\varepsilon_{k}^{d}\right)+G_{k}\left(\gamma_{0},\varepsilon_{k}^{d}\right)\right)} \end{array}$$

Corollary 2 is proven.
